# Electrochemical Corrosion Behavior of Ta_2_N Nanoceramic Coating in Simulated Body Fluid

**DOI:** 10.3390/ma9090772

**Published:** 2016-09-10

**Authors:** Jian Cheng, Jiang Xu, Lin Lin Liu, Shuyun Jiang

**Affiliations:** 1 Department of Material Science and Engineering, Nanjing University of Aeronautics and Astronautics, 29 Yudao Street, Nanjing 210016, China; chengjian@nuaa.edu.cn (J.C.); daicaiyan@gmail.com (L.L.L.); 2 School of Mechanical & Electrical Engineering, Wuhan Institute of Technology, 693 Xiongchu Avenue, Wuhan 430073, China; 3 Department of Mechanical Engineering, Southeast University, 2 Si Pai Lou, Nanjing 210096, China; jiangshy@seu.edu.cn

**Keywords:** Ta_2_N, characterization, mechanical properties, polarization, electrochemical impedance, Mott-Schottky

## Abstract

In order to improve the corrosion and wear resistance of biomedical Ti-6Al-4V implants, a Ta_2_N nanoceramic coating was synthesized on a Ti-6Al-4V substrate by the double glow discharge plasma process. The Ta_2_N coating, composed of fine nanocrystals, with an average grain size of 12.8 nm, improved the surface hardness of Ti-6Al-4V and showed good contact damage tolerance and good adhesion strength to the substrate. The corrosion resistance of the Ta_2_N coating in Ringer’s physiological solution at 37 °C was evaluated by different electrochemical techniques: potentiodynamic polarization, electrochemical impedance spectroscopy (EIS), potentiostatic polarization and capacitance measurements (Mott-Schottky approach). The evolution of the surface composition of the passive films at different applied potentials was determined by X-ray photoelectron spectroscopy (XPS). The results indicated that the Ta_2_N coating showed higher corrosion resistance than both commercially pure Ta and uncoated Ti-6Al-4V in this solution, because of the formed oxide film on the Ta_2_N coating having a smaller carrier density (*N_d_*) and diffusivity (*D_o_*) of point defects. The composition of the surface passive film formed on the Ta_2_N coating changed with the applied potential. At low applied potentials, the oxidation of the Ta_2_N coating led to the formation of tantalum oxynitride (TaO_x_N_y_) but, subsequently, the tantalum oxynitride (TaO_x_N_y_) could be chemically converted to Ta_2_O_5_ at higher applied potentials.

## 1. Introduction

Due to the increased numbers of elderly people in today’s society as a percentage of the population and the growth of accidents, there is ever-increasing demand for artificial joint replacements to surgically substitute for diseased or lost bones to restore form and function [1]. Metallic materials have been extensively used as hard tissue replacements for artificial bone and joint implants. Compared to more conventional stainless steels and cobalt-based alloys, titanium and its alloys have greater advantages for applications in orthopedic implants, because of their lower modulus, lower density, superior biocompatibility and better corrosion resistance [2]. Among various types of Ti alloys, Ti-6Al-4V is the first, and the most commonly used, implant material, particularly for orthopedic prosthesis applications. When implants are placed in the human body, they are in contact with extracellular body fluids, such as blood and interstitial fluids, which not only contain high concentrations of chloride ions, but also amino acids and proteins. Hence, under such an aggressive environment, the corrosion resistance of the metallic implant becomes important, since the corrosion rate is related to the release of metal ions into the tissue surrounding the implant, which, in turn, significantly influences biocompatibility [3]. Corrosion resistance of Ti-6Al-4V in various environments is attributed to the presence of a highly stable and tenaciously adherent passive film spontaneously formed on its surface. This plays a decisive role in determining its corrosion resistance and biocompatibility. However, clinical experience has shown that Ti-6Al-4V is susceptible to localized corrosion in a biological environment, causing the release of metal ions into the tissue adjacent to the implants. The release of metal ions is found to not only be detrimental to bone attachment and further bone growth on the implant surface [4,5], but also causes long-term health problems, such as peripheral neuropathy, osteomalacia and Alzheimer diseases [5,6]. In addition, for load-bearing metallic orthopaedic implants, wear occasionally occurs in cyclic load bearing areas, leading to the generation of debris and the degradation of the passive oxide film integrity to accelerated corrosion damage. Owing to its low hardness, the poor wear resistance of Ti-6Al-4V promotes aseptic loosening after long-term implantation and these abraded debris particles cause inflammatory reaction in the tissue [7].

As is well known, the material surface exerts an important influence on the response of the biological environment to artificial medical devices. Various surface modification techniques [8] have been explored as a means to improve the corrosion and wear resistance of titanium and its alloys, without sacrificing its excellent bulk attributes, such as relatively low modulus, good fatigue strength, formability and machinability. The most effective way to achieve this is to deposit various ceramic coatings, e.g., transition metal oxides, nitrides, carbides, or silicides [9,10,11]. These can provide a barrier layer between the bioenvironment and the alloy substrate. Recently, due to their high thermal stability, stable electrical resistivity, wear resistance and superior corrosion, tantalum nitrides have received much attention as protective coating materials and have been applied in a range of mechanical and microelectronic industries, including application as hard coatings for cutting tools [12], diffusion barrier layers in integrated circuits [13], and stable thin film resistors [14]. Further, similar to metallic Ta, tantalum nitride based materials (for instance, TaN, Ta_2_N and TaC_x_N_1−x_) have enormous potential in biomedical fields. Leng et al. found that tantalum nitride films showed better blood compatibility than low-temperature isotropic pyrolytic carbon (LTIC), and suggested that they could be a possible alternative for LTIC in artificial heart valves [15]. Nonetheless, data on the electrochemical behavior of tantalum nitride in physiological solutions are still scarce. In the present work, a novel Ta_2_N nanoceramic coating was prepared on Ti-6Al-4V substrate by a double glow discharge plasma process. The microstructure of the Ta_2_N nanoceramic coating was characterized by X-ray diffraction (XRD), scanning electron microscopy (SEM) and transmission electron microscopy (TEM). Hardness and adhesion strength of the coating to the substrate were evaluated using nanoindentation and scratch testing, respectively. To evaluate its potential use in biomedical applications, the electrochemical behavior of the Ta_2_N coating was examined in Ringer’s solution by various electrochemical methods including potentiodynamic polarization, potentiostatic polarization, EIS and Mott-Schottky analysis. The corrosion resistance of commercially pure Ta and uncoated Ti-6Al-4V was also measured to provide a comparative study.

## 2. Materials and Methods

### 2.1. Coating Specimen Preparation

The samples were cut from a medical grade Ti-6Al-4V alloy bar. The Ti-6Al-4V substrates were abraded with 2000-grit SiC paper, polished with 0.5 μm Cr_2_O_3_ powder and then cleaned using double-distilled water. The sputtering target is a 99.99% pure Ta disk with a diameter of 100 mm and thickness of 5 mm. The Ta_2_N coating was fabricated on a Ti-6Al-4V substrate using a double cathode glow discharge apparatus. During the preparation of coating samples, one cathode served as the sputtering target, and the other cathode is the substrate material, as described in detail elsewhere [10]. The base pressure in the chamber was evacuated down to 5 × 10^−4^ Pa. Prior to deposition, the substrate surface was cleaned by argon ion sputtering for 10 min to remove surface contamination at about 20 Pa and a substrate bias voltage of −600 V. During deposition, the working pressure of 35 Pa is composed of Ar and N_2_ gas mixture, with N_2_:Ar flux ratio of 1:20. The sputtering parameters of deposition used here include: the target electrode bias voltage of −900 V, substrate bias voltage of −350 V; substrate temperature of 850 °C, a parallel distance between the target and the substrate of 10 mm and a deposition time of 1.5 h.

### 2.2. Phase and Microstructural Characterization

The crystal structure of the as-deposited coating was analyzed by a D8 ADVANCE X-ray diffractometer using Cu K*_α_* radiation. The surface and cross-sectional morphology of the as-prepared coating were determined using a S-4800 field emission scanning electron microscopy. TEM observations were performed using a JEOL JEM-2010 transmission electron microscope operating at an accelerating voltage of 200 kV. X-ray photoelectron spectroscopy (XPS) measurements were carried out on a Kratos AXIS Ultra ESCA system using Al Ka (1486.6 eV, pass energy of 20 eV). The deconvolution of the spectra was achieved by fitting the data using the software XPS PEAK4.1. Peak identification was performed with reference to an XPS database.

### 2.3. Mechanical Properties Measurements

Nanoindentation tests were conducted on the Ta_2_N coating and Ti-6Al-4V substrate using a nanoindentation tester (Ultra-Micro Indentation System 2000), equipped with a Berkovich diamond tip. The standard analysis procedure proposed by Oliver and Pharr [16] was used to determine the hardness of the samples from the load–displacement curves. The adhesion strength of the Ta_2_N coating to Ti-6Al-4V substrate was evaluated by a WS-2005 scratch tester equipped with an acoustic emission (AE) detector. During the scratch tests, a 200 μm radius Rockwell C diamond indenter was drawn across the coating surface under a linearly increased normal load from 0 N to 100 N with a constant loading rate of 20 N/min. The contact-damage resistance of the coating was assessed using an applied normal load of 9.80 N with a Vickers indenter. A focused ion beam (FIB) milling system (Nova Nanolab 200, FEI, Hillsboro, OR, USA) was used to obtain subsurface sectioning and imaging of indentation sites.

### 2.4. Electrochemical Measurements

The electrochemical measurements were conducted using a CHI604D electrochemical workstation (Shanghai ChenHua Instruments Co., Shanghai, China) in a three-electrode configuration. The test specimen, a platinum plate and a saturated calomel electrode (SCE) were used as the working, auxiliary, and reference electrodes, respectively. The work electrolyte was Ringer’s physiological solution, which contains 8.61 g of NaCl, 0.49 g of CaCl_2_ and 0.30 g of KCl diluted in 1 L of water. Ringer’s solution was naturally aerated and maintained at 37 ± 0.5 °C to simulate the body temperature. Prior to electrochemical experiments, working electrodes were initially reduced potentiostatically at −0.8 V for 10 min to remove air-formed oxides. The polarization curves were recorded potentiodynamically at a scan rate of 20 V/min from −0.5 to 1.5 V. Electrochemical impedance spectroscopy (EIS) measurements were conducted over the frequency from 100 kHz to 10 mHz with a 10 mV amplitude signal at open circuit potential (E_OCP_). ZSimpWin software was used to fit the appropriate electrical equivalent circuit (EEC) model into the EIS experimental data. In order to characterize the electronic properties of the passive films, steady-state passive films were formed by potentiostatically polarizing the specimens at different film formation potentials (E_f_, 0.4 V, 0.6 V, 0.8 V and 1.0 V) for 60 min. The Mott-Schottky analysis were performed by sweeping the potential from E_f_ in the negative direction with potential steps of 25 mV, at a frequency of 1 kHz with an amplitude signal of 10 mV. Uncoated Ti-6Al-4V and commercially pure Ta (99.99% pure) were used as reference materials for the electrochemical measurements.

## 3. Results and Discussion

### 3.1. Microstructure and Phase Analysis

**Figure 1:**
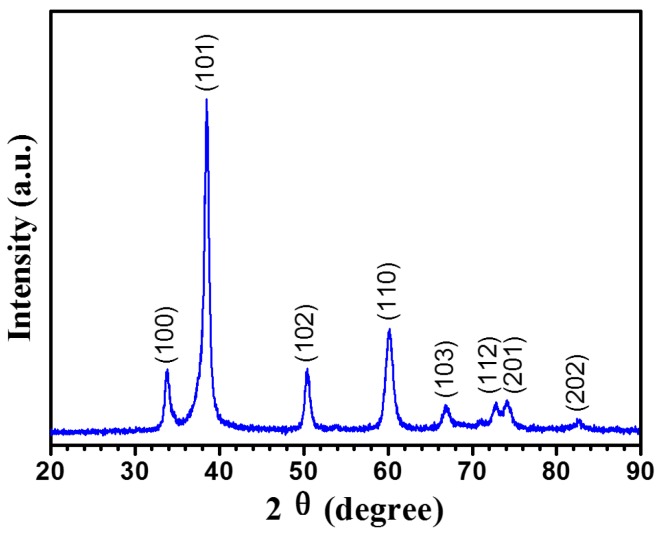
Typical X-ray diffraction pattern taken from the as-deposited Ta_2_N coating.

**Figure 2:**
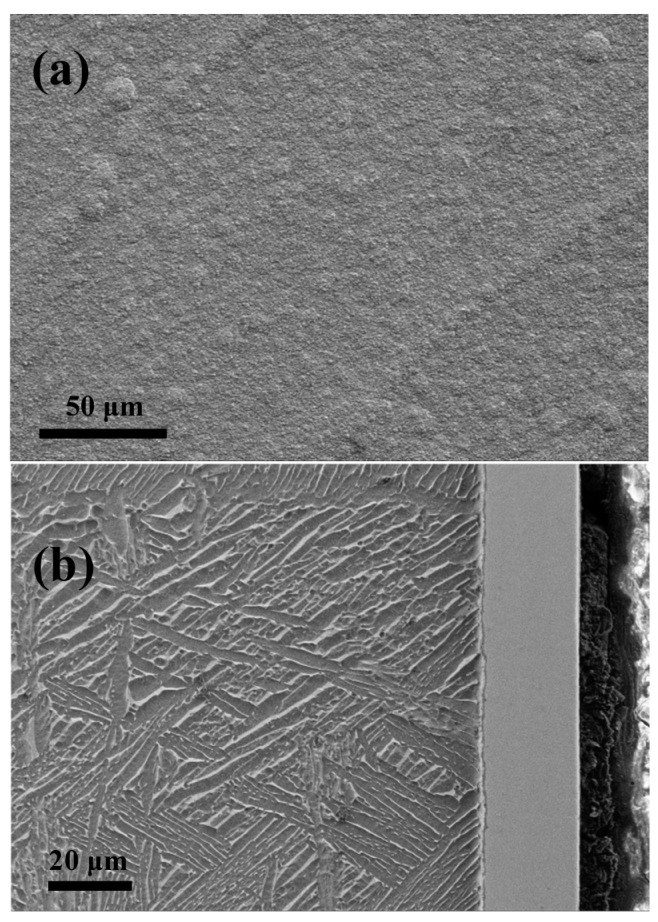
(**a**) Plan view and (**b**) cross-sectional SEM morphologies of the Ta_2_N coating.

A typical X-ray diffraction pattern recorded from the Ta_2_N coating is shown in Figure 1. All the diffraction peaks acquired from the as-deposited coating can be indexed to the hexagonal structured Ta_2_N phase based on data in JCPDS card No. 26-0985. No other characteristic peaks were detected, indicating that the coating consists of single phase Ta_2_N. In addition, clear diffraction peak broadening with relatively low peak intensity can be observed in the pattern, suggesting that the coating contains very fine grains. Scherrer analysis of the X-ray diffraction pattern give an average crystal size of ~10.5 nm from the (101) plane, after correction for instrumental line broadening. Figure 2a,b show, respectively, representative plan view and cross-sectional SEM images for the Ta_2_N coated Ti-6Al-4V. As shown in Figure 2a, the Ta_2_N coating has a relatively smooth and uniform surface. The SEM cross-sectional view (Figure 2b) of the Ta_2_N coated Ti-6Al-4V reveals that the Ta_2_N coating, with an average thickness of ~25 μm, is of good quality without any visible defects and tightly adhered to the Ti-6Al-4V substrate.

**Figure 3:**
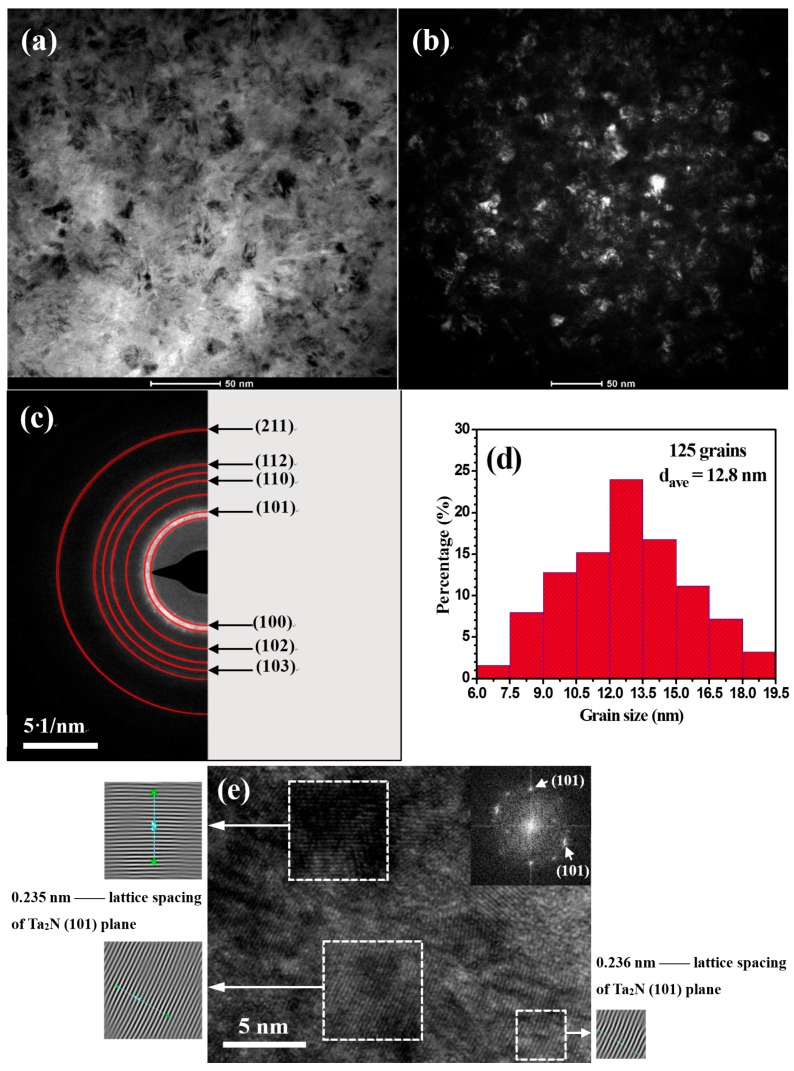
(**a**) Bright-field and (**b**) dark-field plan-view TEM images; (**c**) corresponding selected area electron diffraction (SAED) pattern; (**d**) statistical histogram of the Ta_2_N grain sizes and (**e**) high resolution TEM image for the as-deposited Ta_2_N coating.

A bright-field/dark-field pair, Figure 3a,b, show clearly that the microstructure of the coating is composed of uniformly distributed fine nanocrystals with irregular morphologies. The Debye-Scherrer rings in the selected area diffraction (SAD) pattern (Figure 3c) from the inside to outside are assigned to the hexagonal structured Ta_2_N (100), (101), (102), (110), (103), (112) and (211) planes, respectively, which is in complete agreement with the XRD results. Figure 3d shows the grain size distribution based on the analysis of more than 120 grains (Figure 3d). The results indicate that the majority of the grains have a grain size smaller than 15 nm and the average grain size based on statistical analysis is 12.8 nm. It is worthwhile to note that the average grain size estimated by TEM is slightly larger than the grain size measured by XRD, because the XRD data neglect contributions from structural defects (i.e., stacking faults or low-angle grain boundaries) and microstraining [17]. From the bright-field high-resolution TEM (HRTEM) image shown in Figure 3e, the nanocrystallites have clear lattice fringes and interplanar distance of the crystallites marked with a white frame was calculated to be ~0.235 nm, corresponding to the d-spacing of the {101} planes of hexagonal Ta_2_N.

### 3.2. Mechanical Properties

**Figure 4:**
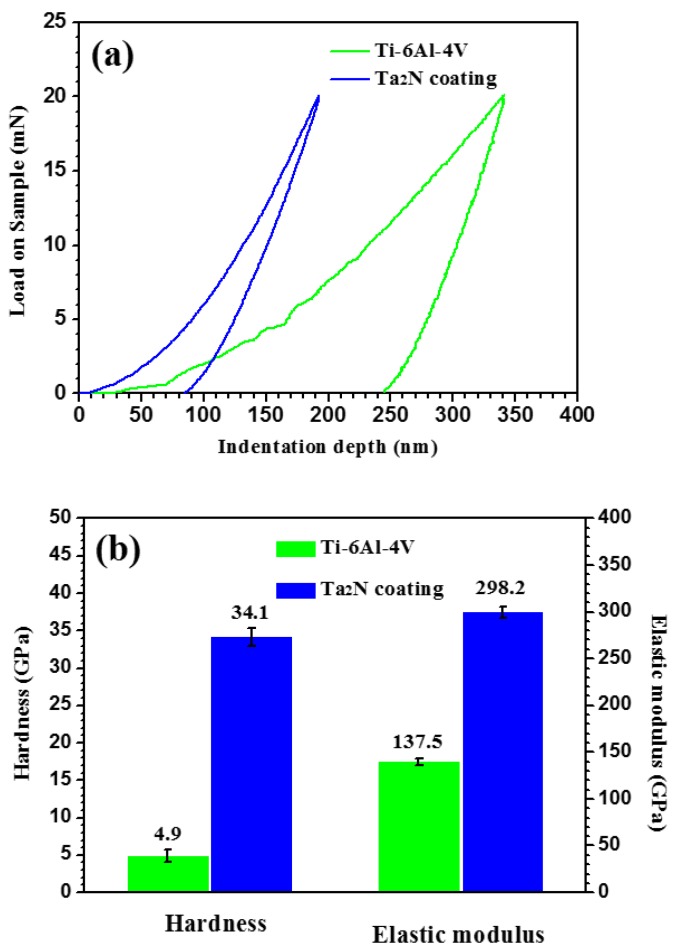
(**a**) Load-displacement curves and (**b**) hardness and elastic modulus for the Ta_2_N coating and uncoated Ti-6Al-4V.

Typical load-displacement curves for the performed nano-indentation tests for both the Ta_2_N coating and uncoated Ti-6Al-4V are plotted in Figure 4a. The curve for the Ta_2_N coating is continuous, indicating no occurrence of any abrupt cracking of the coating. Under the same loading conditions, the residual indentation depth and maximum indentation depth for the Ta_2_N coating are shallower than those for uncoated Ti-6Al-4V, denoting that the Ta_2_N coating possesses higher resistance to local plastic deformation. The elastic recovery (ER) is defined as ER = (d_max_ − d_res_)/d_max_, where d_max_ and d_res_ are the displacement at the maximum load and residual displacement after unloading, respectively. It can be found that the elastic recovery values increase from 28% for uncoated Ti-6Al-4V to 55% for the Ta_2_N coating, suggesting better elastic resistance for the Ti-6Al-4V substrate coated with Ta_2_N. As shown in Figure 4b, the hardness and elastic modulus of the Ta_2_N coating were determined to be 34.1 ± 0.9 GPa and 298.2 ± 6.6 GPa, respectively, which are approximately equivalent to the reported values for a Ta_2_N coating prepared by radio-frequency (RF) magnetron sputtering [18]. Additionally, the ratio of the maximum indentation depth to the coating thickness is less than 10%, suggesting that the contribution to mechanical properties from the substrate can be negligible.

**Figure 5:**
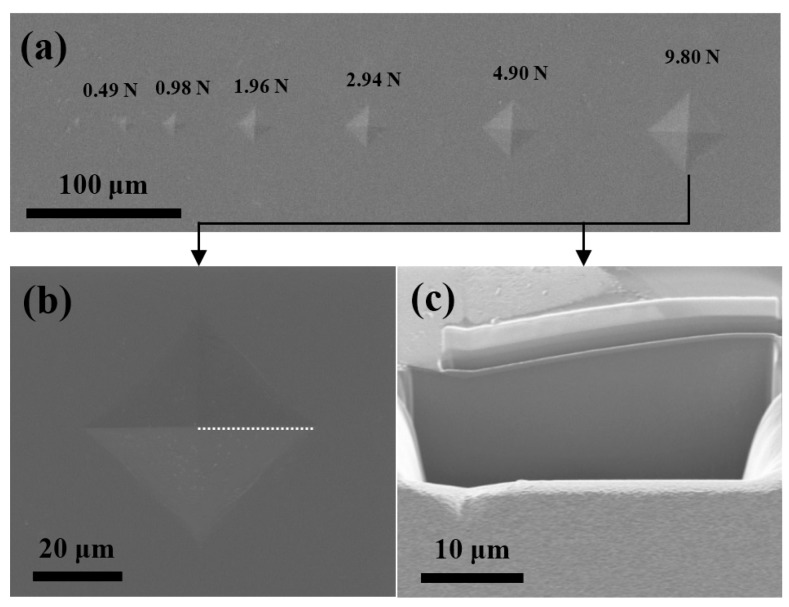
(**a**) Optical micrographs of Vickers indentations in the Ta_2_N coating at applied loads ranging from 0.49 to 9.80 N; (**b**) Plan view and (**c**) cross-sectional secondary electron FIB images of Vickers indentation under an indentation load of 9.80 N.

The contact damage resistance of the Ta_2_N coating was further evaluated using the Vickers indentation method under various loads. Figure 5a shows optical micrographs of Vickers indentations in the Ta_2_N coating. Careful observation of the Vickers indentations, obtained at loads ranging from 0.49 to 9.80 N, reveals that indentations obtained at these various loads are nearly ‘perfect’, with no evidence of microcracking at the corners of the indent, where the coating undergoes the highest tensile stress. Similarly, no additional cracking is visible within cross-sections taken from the plastically deformed region below the indent obtained at a load of 9.80 N (Figure 5c). The above results suggest that the Ta_2_N coating exhibits good contact damage tolerance.

**Figure 6:**
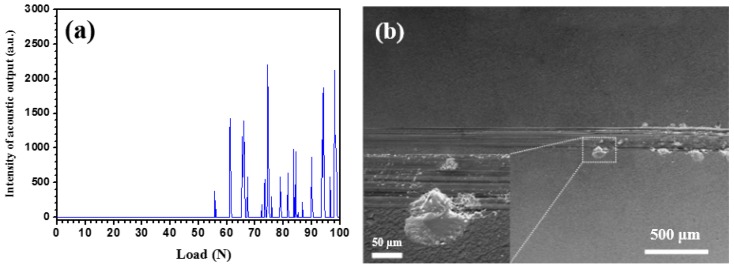
(**a**) Acoustic emission signal peaks versus normal load curve and (**b**) SEM image of the scratch track for the Ta_2_N coating.

The adhesion strength of a coating on a metallic implant is one of the most important properties for orthopedic applications, since the interfacial adhesive strength of the coating affects the lifetime of a biomaterial [19]. The scratch test is commonly performed to evaluate the adhesion strength of a coating/substrate. Figure 6a shows the acoustic emission signals measured as a function of normal load. As shown in Figure 6a, once the normal load has reached a critical value of ~56 N, continuous acoustic emission peaks were generated. From the scratch track evident in the SEM image (Figure 6b), both the width of the scratch track and the extent of the surface damage increase with increasing normal load, and when the normal load reaches to the critical value, a large area of delamination is evident at the edge of the scratch track. As a general rule, a critical load of above 30 N measured with a Rockwell C diamond tip during scratch testing is believed to be sufficient for most engineering applications [20]. Therefore, the Ta_2_N coating possesses sufficient adhesion strength to meet long-term use in the human body under load-bearing conditions.

### 3.3. Open Circuit Potential Measurements

**Figure 7:**
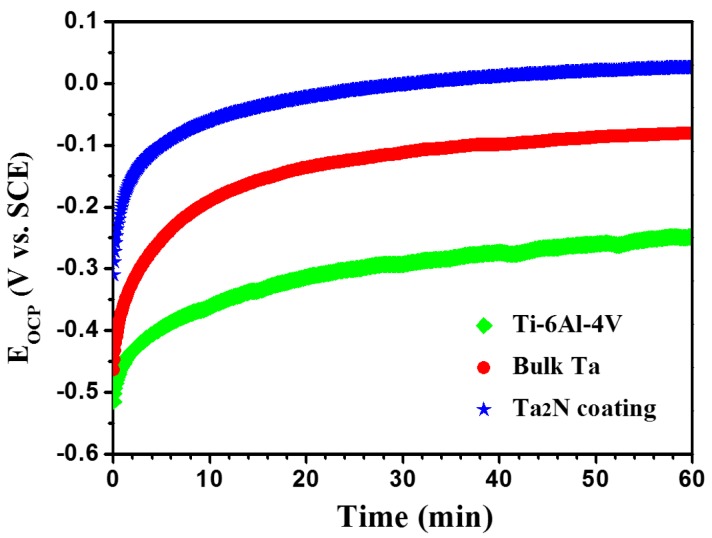
Variation of open circuit potential with time for uncoated Ti-6Al-4V, commercially pure Ta and the Ta_2_N coating in Ringer’s physiological solution at 37 °C.

Figure 7 presents the variation of the open circuit potential (E_OCP_) for the Ta_2_N coating, commercially pure Ta and uncoated Ti-6Al-4V as a function of immersion time in Ringer’s physiological solution at 37 °C. As can be seen, similar E_OCP_ evolution behavior is observed for all specimens. E_OCP_ shifts quickly towards more positive potentials during the first 10~15 min. After that, E_OCP_ changes more slowly until it reaches a quasi-stationary value. Such E_OCP_ evolution behavior suggests that all specimens undergo spontaneous passivation due to spontaneously formed oxide film passivating the specimen surface in the Ringer’s physiological solution, which is typical of passivated materials. Figure 7 also shows a significant ennoblement in the E_OCP_ of the Ta_2_N coating compared with that of uncoated Ti-6Al-4V, indicating that the Ta_2_N coating has higher thermodynamic stable oxide film.

### 3.4. Potentiodynamic Polarization Tests

**Figure 8:**
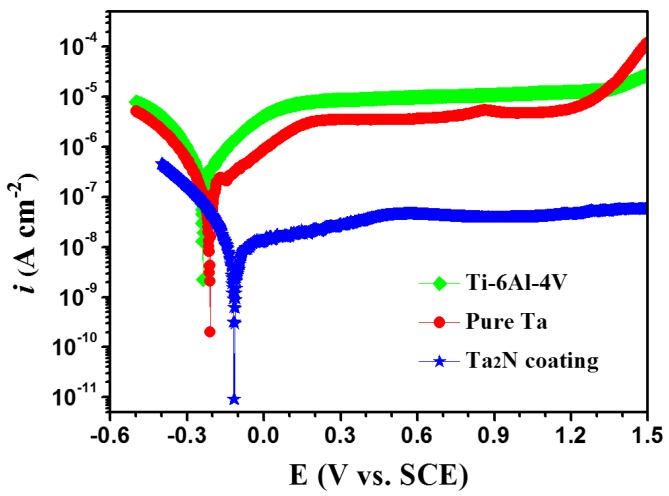
Potentiodynamic polarization curves of uncoated Ti-6Al-4V, commercially pure Ta and the Ta_2_N coating in Ringer’s physiological solution at 37 °C.

Figure 8 shows typical potentiodynamic polarization plots recorded from the Ta_2_N coating, commercially pure Ta and uncoated Ti-6Al-4V exposed to naturally aerated Ringer’s physiological solution at 37 °C. The corresponding electrochemical parameters derived from the polarization curves are summarized in Table 1. It can be seen from Figure 8 that the shape of the polarization curves is quite similar for the three tested samples. The cathodic Tafel slopes (-*β*_c_) of the tested specimens exhibit values in the range from 116.48 to 120.63 mV/decade, which indicates that the reduction of dissolved oxygen and water takes places on the samples’ surfaces, due to fact that the measurements were carried out in the aerated condition [21]. From the inspection of the anodic branch of the polarization plots, all of the tested samples show wide passive region, in where the passive current densities are almost independent of any increase in potential. The larger anodic Tafel slopes (*β*_a_) value in comparison with the *β*_c_ value indicates anodic control in the corrosion process of the three tested samples [22], implying that the existence of the protective oxide films gives rise to a typical passive character of the tested samples with a low corrosion current density. The corrosion potential for the Ta_2_N coating was found to be nobler than for both commercially pure Ta and uncoated Ti-6Al-4V, signifying that the Ta_2_N coating possess a higher electrochemical inertness in such an environment. Generally, the corrosion current density (*i*_corr_) is an important parameter to evaluate the kinetics of corrosion reactions and corrosion rate is proportional to the corrosion current density measured via polarization [23]. In other words, the lower the *i*_corr_, the lower the corrosion rate. For the Ta_2_N coating, the corrosion current density is very low, of the order of magnitude of about 10^−9^ A·cm^−2^, which is two orders of magnitude less than those of the two reference samples. Similarly, the current density within the passive range for a potential of 0.6 V is 4.55 × 10^−8^ A·cm^−2^ for the Ta_2_N coating, which is also two orders of magnitude smaller than for uncoated Ti-6Al-4V and commercially pure Ta, suggesting that the passive film formed on the Ta_2_N coating has higher electrochemical stability. Furthermore, defects (i.e., pores and/or pinholes) present in coatings are assumed to connect the bulk electrolyte to the substrate surface and may result in severe localized corrosion due to the galvanic effect when the coating is anodic with respect to the substrate. Assuming that the Ta_2_N coating is electrochemically inert at low anodic overpotentials, the porosity of the coating (P) can be estimated from the polarization resistance, *R_p_*, using Equation (1) [24]:

(1) $$P=\frac{R_{ps}}{R_{pc}}\times 10^{-\left|△E_{corr}\right|/\beta_{a}}$$

where *R_ps_* and *R_pc_* are the measured polarization resistance of substrate and coating, respectively, ∆*E_corr_* is the difference of the corrosion potential between substrate and coating, *β_a_* is the anodic Tafel slope for the substrate. The polarization resistance is obtained from the potentiodynamic polarization curves using Equation (2) [25]:

(2) $$R_{p}=\frac{\beta_{a}\beta_{c}}{2.303\left(\beta_{a}+\;\beta_{c}\right)i_{corr}}$$

where *β*_a_ and *β*_c_ are, respectively, the anodic and cathodic Tafel slopes, and *i*_corr_ is the corrosion current density (A·cm^−2^). The calculated porosity value of the Ta_2_N coating is 0.22%, confirming that the number of defects in the coating is low. Low porosity may be related to its thickness, since porosity of a coating is inversely proportional to coating thickness.

### 3.5. Electrochemical Impedance Spectroscopy (EIS) Measurements

**Figure 9:**
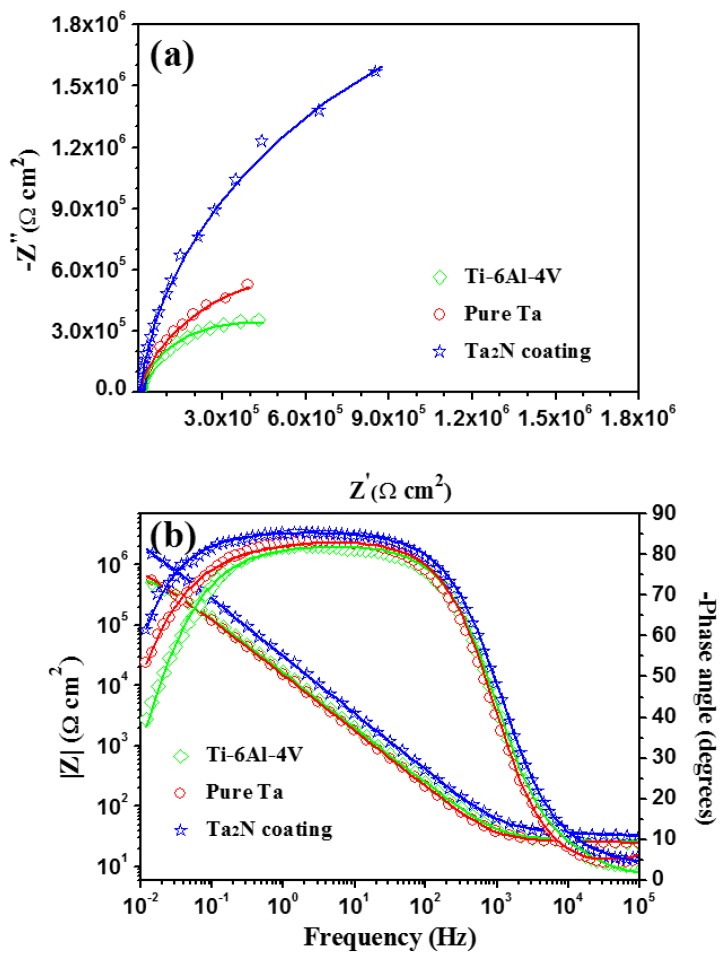
(**a**) Nyquist and (**b**) Bode plots of uncoated Ti-6Al-4V, commercially pure Ta and the Ta_2_N coating at respective open circuit potentials in Ringer’s physiological solution at 37 °C. Symbols are experimental data and solid lines are fitted results.

The impedance spectra recorded at the open-circuit potentials (OCPs) for the Ta_2_N coating, commercially pure Ta and uncoated Ti-6Al-4V in naturally aerated Ringer’s physiological solution at 37 °C are displayed in the form of both Nyquist plots, and Bode amplitude and phase angle plots in Figure 9. The Nyquist plots for the tested samples show similar features, i.e., the response of the Nyquist complex plane are characterized by a unique capacitive loop with a wide incomplete semicircle over the whole range of frequency, typical behavior for passive materials [26]. It is generally accepted that the larger the diameter of the semicircle, the better the corrosion resistance of the sample. It is evident from Figure 9a that the diameter of the capacitive semicircle of the Ta_2_N coating is markedly larger than those of the two reference samples, indicating a higher corrosion resistance for the coating. In addition, the Z_imag_/Z_re_ ratio of the Ta_2_N coating is also greater than those of the two reference samples, indicating an enhanced capacitive behavior for the solid/liquid interface. As shown in Figure 9b, the corresponding Bode-phase plots for the tested specimens are similar in shape with high symmetry. In the high frequency region, the log|Z| is almost frequency-independent with the phase angle falling rapidly towards zero with increasing frequency, indicative of a representative response of the electrolyte resistance. In the intermediate frequency region, a linear relationship between the log |Z| and log *f* is observed with a slope approaching −1 and a phase angle close to −90°, especially in the case of the Ta_2_N coating. This is a typical capacitive response, associated with the presence of passive films on the tested samples acting as an efficient barrier to corrosion attack. In the low frequency region, the phase angles for the tested samples shift to the lower values suggesting the decreased capacitive influence in the electrochemical behavior of the samples. Both the impedance modulus at the lowest frequency (|Z|*_f_*_→0_) and the phase angle maximum for the Ta_2_N coating are bigger than those of the two reference samples, indicating better protective properties of the oxide film formed on the coating.

**Figure 10:**
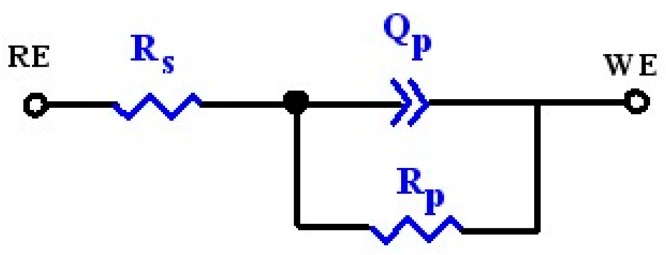
Electronic equivalent circuit (EEC) used in the fitting procedure of the EIS experimental data.

To gain better insight regarding the corrosion occurring on the samples, EIS spectra were analyzed using equivalent electric circuits shown in Figure 10. As shown in Figure 9, the impedance spectra of the tested samples exhibit only one time constant, indicating that the sample-electrolyte interface could be modeled by a resistance *R_p_* and a constant phase element (CPE, *Q_p_*) in parallel, which is connected in series with the solution resistance (*R_s_*). In this circuit, a constant phase element (CPE, *Q_p_*), instead of pure capacitance, is used to obtain a satisfactory fit. The physical origin of the CPE behavior has been widely discussed in the literature, and it is generally believed to originate from current and potential heterogeneous distributions due to surface disorder and roughness [27]. The impedance of Z_CPE_ is defined as Z_CPE_ = [Q(*jω*)^n^]^−^^1^, where *Q* is the CPE constant (Ω^−^^1^·cm^−^^2^·s^n^), *ω* is the angular frequency (rad·s^−^^1^), *j^2^* = −1 is an imaginary number, and *n* is the CPE exponent. Using the proposed equivalent circuits, a good match between the measured and simulated data was achieved for each spectrum. The typical chi-square (*χ*^2^) values varied from 5.61 × 10^−4^ to 9.35 × 10^−4^, indicating a satisfactory fit. Table 2 compiles numerical values of the circuit elements from the fitting procedure. From Table 2, the resistance value (*R_p_*) of the Ta_2_N coating is three times greater than that of commercially pure Ta and one order of magnitude higher than that of uncoated Ti-6Al-4V. The resistance (*R*) values were highly dependent on the solution in which the measurements were made, whereas the capacitance (*C*) values are independent of the solution used and thus can provide information about the dielectric property of the passive film [28]. The effective capacitance (*C_p_*) can be calculated from the proposed equivalent circuit model with a CPE using the expression developed by Brug et al. [29]

(3) $$C_{p}=Q_{p}{}^{1/n}{\left(R_{s}{}^{-1}+R_{p}{}^{-1}\right)}^{\left(n-1\right)/n}$$

The *C_p_* values for the Ta_2_N coating, commercially pure Ta and uncoated Ti-6Al-4V are 3.35, 6.19 and 8.43 μF·cm^−^^2^, respectively. Higher resistance, combined with lower capacitance, endows the Ta_2_N coating with higher insulating or dielectric property as compared to commercially pure Ta and uncoated Ti-6Al-4V. This is consistent with the result of the polarization tests. In addition, the *n* value of the Ta_2_N coating is greater than those of both commercially pure Ta and uncoated Ti-6Al-4V, reflecting that the passive film formed on the Ta_2_N coating is more homogeneous and compact than those formed on the two reference samples [30]. The time constant (*τ*) describing the charge transport process in the passive layer can be expressed as *τ* = *C* × *R*. This parameter can be directly used to evaluate the rate of relevant electrochemical process, including ionic migration within the oxide film and charge transfer [31,32]. According to the results shown in Table 2, *τ* values of the tested samples increase in the order of uncoated Ti-6Al-4V < commercially pure Ta < the Ta_2_N coating. About a three times enhancement in time constant is achieved by implementation of a Ta_2_N coating on the Ti-6Al-4V substrate, indicating that the Ta_2_N coating can effectively retard the electrochemical corrosion process.

### 3.6. Potentiostatic Polarization Tests

**Figure 11:**
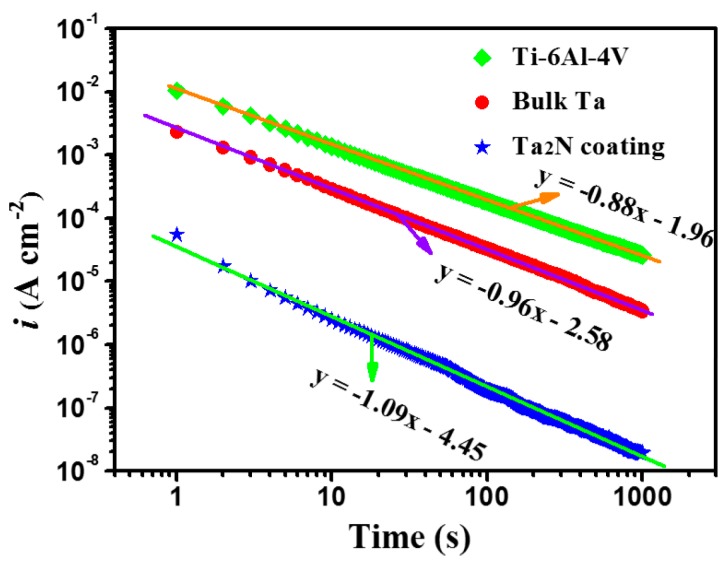
Double-log plots of current-time for uncoated Ti-6Al-4V, commercially pure Ta and the Ta_2_N coating potentiostatically polarizated at 0.8 V in Ringer’s physiological solution.

The compactness is one of the key factors that influence the stability of the passive film and can be assessed by potentiostatic polarization analysis. Assuming the contribution of the double layer charge is negligible, the initial drop in current density should be related to the growth of the passive film on the electrode surface after cathodic reduction [33]. The current decreased with time according to the formula [34]:

(4) $$i=10^{-\left(A+klgt\right)}$$

where *k* represents the slope of the double-log plot for potentiostatic polarization, *i* is the current density, *t* is time and *A* is constant. *k* = −1 usually signifies the formation of a compact, highly protective passive film, while *k* = −0.5 indicates the formation of a porous film growing from a dissolution and precipitation process. Figure 11 shows the double-log plots of potentiostatic current-time transients recorded at 0.8 V for the Ta_2_N coating, commercially pure Ta and uncoated Ti-6Al-4V in Ringer’s physiological solution at 37 °C. The values of *k* for the tested specimens increases in the following sequence: uncoated Ti-6Al-4V < commercially pure Ta < the Ta_2_N coating, implying the increased compactness of the passive films formed on the tested samples. Hence, the passive film formed on Ta_2_N coating with higher compactness exhibits a greater protective ability to hinder the penetration of corrosive species into the underlying film, as compared to the two reference samples.

### 3.7. XPS Analysis of Passive Film Composition

**Figure 12:**
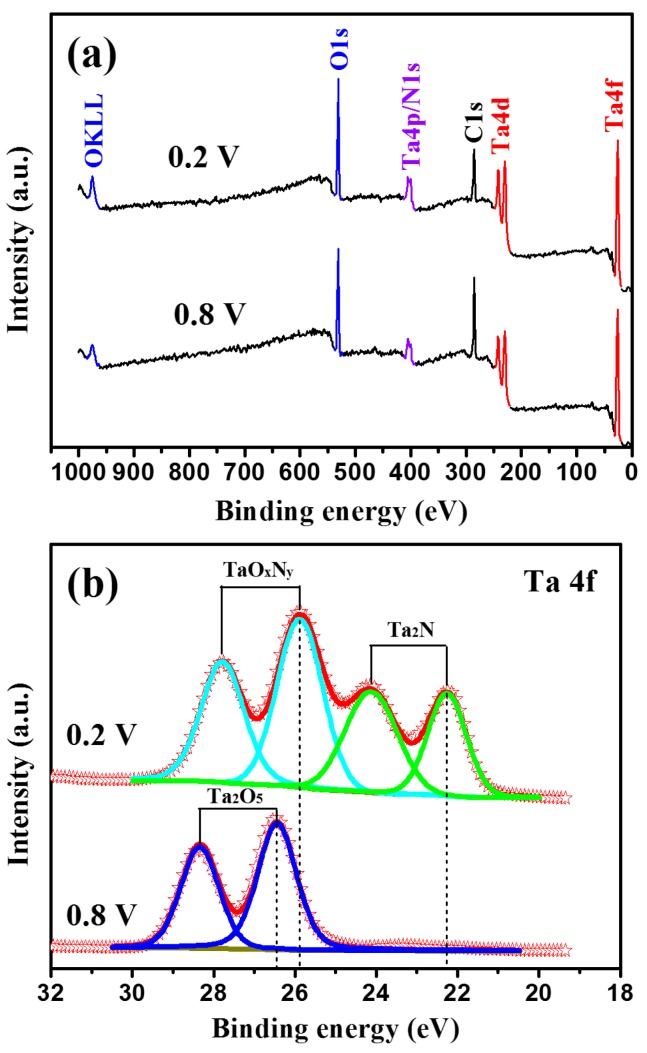

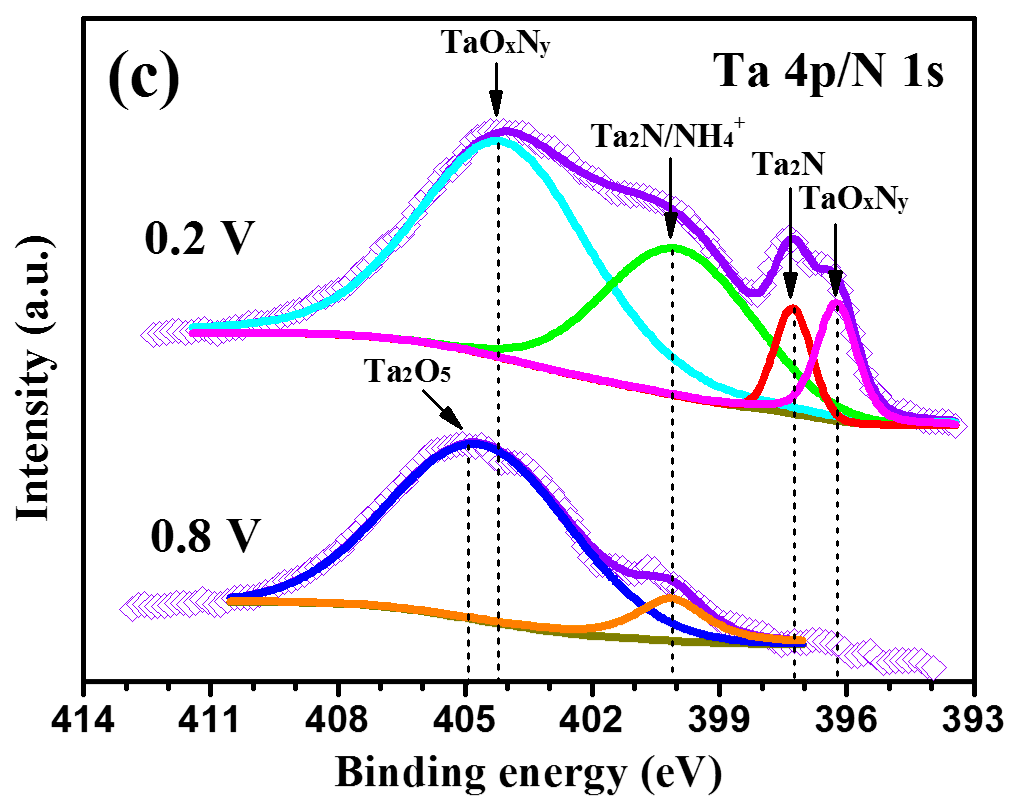
(**a**) XPS survey spectra and (**b**,**c**) high-resolution XPS spectra for Ta 4f and Ta 4p/N 1s for the passive films formed on the Ta_2_N coating after potentiostatic polarization at 0.2 V and 0.8 V for 60 min in Ringer’s physiological solution.

In order to gain insight into the change in the composition and chemical state of the elements of the passive films at different applied potentials, XPS analysis was performed ex situ on the Ta_2_N coating after potentiostatic polarization at 0.2 V and 0.8 V for 60 min in Ringer’s physiological solution at 37 °C (Figure 12). As shown in Figure 12a, the XPS survey spectra of the two passive films grown on the Ta_2_N coating exhibit peaks from Ta, O, N and C. The C1s peak possibly arises from a contaminant hydrocarbon layer covering the outermost surface of the specimen. Figure 12b,c show high-resolution XPS spectra for Ta 4f and Ta 4p/N 1s core levels regions obtained from the two passive films formed on the Ta_2_N coating, respectively. After deconvolution, the Ta 4f spectrum (Figure 12b) recorded from the passive film formed at 0.2 V presents two sets of doublets of Ta 4f_7/2_ and Ta 4f_5/2_. One doublet at the low-energy-side with a binding energy of Ta 4f_7/2_ at 22.3 eV can be assigned to Ta_2_N [35], while another high-energy-side doublet with a binding energy of Ta 4f_7/2_ positioned at 25.9 eV can be attributed to TaO_x_N_y_ [36]. Note that the detection of Ta_2_N may possibly originate from the Ta_2_N coating underneath the passive film. In the case of the passive film formed at 0.8 V, the Ta 4f core level shows one doublet with a binding energy of Ta 4f_7/2_ located at 26.4 eV, which corresponds to the binding energy of Ta in stoichiometric Ta_2_O_5_ [37]. In Figure 12c, XPS spectra for the Ta 4p_3/2_/N 1s region obtained from the passive film formed at 0.2 V can be deconvoluted into four peak components. Two peaks located at binding energies of 404.2 eV and 396.6 eV can be assigned to the Ta 4p_3/2_ peak and the N 1s peak of TaO_x_N_y_ [36]. Another N 1s peak at a binding energy of 397.2 eV is characteristic of the Ta-N bond in Ta_2_N [36]. The component with a binding energy of 400.1 eV corresponds to the N 1s binding energy of N–H in NH_4_^+^ [38]. Compared to that for TaO_x_N_y_, the Ta 4p_3/2_ peak recorded from the passive film formed at 0.8 V is shifted to a higher binding energy by 0.8 eV, which is typical of the Ta chemical states in stoichiometric Ta_2_O_5_ [37]. The shift in binding energies for Ta 4p_3/2_ is attributed to the covalency between Ta and O being less than between Ta and N, because nitrogen is less electronegative than oxygen [39]. In addition, the peak at a binding energy of 400.1 eV was also found on the passive film formed at 0.8 V. According to the principal constituent of the passive films formed on the Ta_2_N coating, two possible hydrolysis reactions can be proposed to describe the chemical interaction of the Ta_2_N coating with Ringer’s physiological solution at different applied potentials:

(5) $${\mathrm{Ta}}_{2}N+H_{2}O\to {\mathrm{TaO}}_{x}N_{y}+{\mathrm{NH}}_{4}{}^{+}+{\mathrm{OH}}^{-}$$

(6) $${\mathrm{TaO}}_{x}N_{y}+H_{2}O\to {\mathrm{Ta}}_{2}O_{5}+{\mathrm{NH}}_{4}{}^{+}+{\mathrm{OH}}^{-}$$

Firstly, at a low applied potential of 0.2 V, the oxidation of the Ta_2_N coating leads to the formation of a tantalum oxynitride (TaO_x_N_y_) passive film on the coating surface by Reaction (5), and subsequently the tantalum oxynitride (TaO_x_N_y_) can be chemically converted by oxidation (Reaction (6)) into Ta_2_O_5_ at a higher applied potential of 0.8 V.

### 3.8. Mott-Schottky Analysis and Point Defect Model

In general, the protective films formed on passivated materials, regarded as defective oxide layers, are known to exhibit the semiconducting properties, which have been correlated with their electrochemical behavior [40]. Mott-Schottky analysis, through measuring of the electrode capacitance as a function of applied potential, has been proven to be a powerful tool for providing precise information on the electronic properties of the passive films [41]. According to the Mott-Schottky theory [42], under the depletion conditions, the linear relation between the reciprocal of the square of the space charge capacitance (C_sc_) and the applied potential (E) can be expressed as:

(7) $$\frac{1}{C_{sc}{}^{2}}=\frac{1}{C^{2}}=\frac{2}{\varepsilon_{r}\varepsilon_{0}qN_{q}}\left(E-E_{fb}-\frac{kT}{q}\right)$$

where *ε_r_* is the dielectric constant of the passive film (~60 [41] for TiO_2_ and ~25 [43] for Ta_2_O_5_), *ε*_0_ is the vacuum permittivity (8.854 × 10^−14^ F·cm^−1^), *q* is the elementary charge (+e for electrons and –e for holes), *N_q_* is the density of charge carriers (*N_d_* for donors and *N_a_* for acceptors), *E* is the applied potential, *E_fb_* is the flat band potential, *k* is the Boltzmann constant (1.38 × 10^−23^ J·K^−1^), and *T* is absolute temperature (310 K). The term *kT/q* can be neglected since it is only ~27 mV at 310 K. The measured interfacial capacitance, *C*, is obtained from *C* = −*1/ωZ"*, where *ω* = *2πf* is the angular frequency and *Z"* is the imaginary part of the impedance. The Mott-Schottky analysis is based on the assumption that the contribution of the Helmholtz double-layer capacitance (C_H_) can be neglected and the capacitance of the passive films is mainly dominated by the *C_SC_*. In our case, this assumption is valid since the measured capacitances on the tested specimens (as shown in Table 2) are far less than the values of C_H_ reported in the literature (about 20~50 μF·cm^−2^ [44,45]).

**Figure 13:**
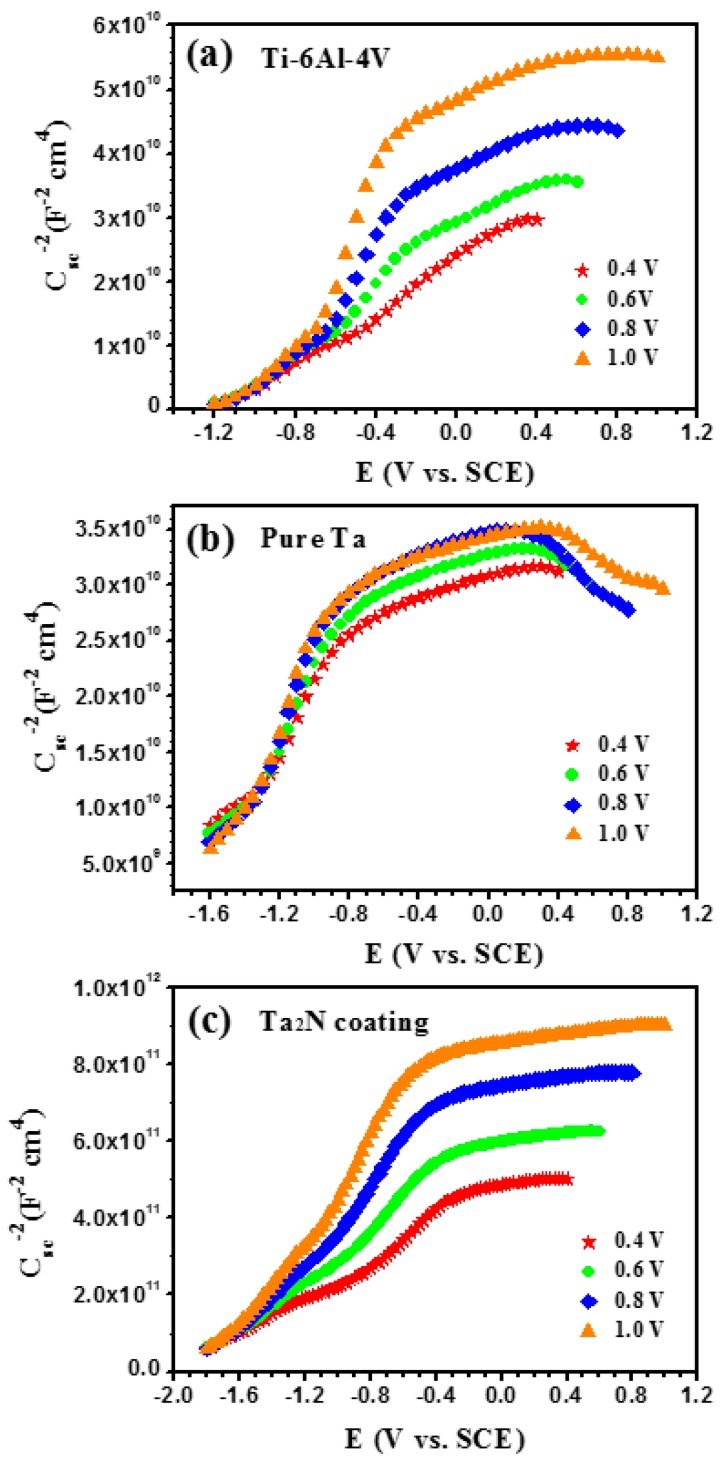
Mott-Schottky plots of the passive films formed at different potentials (0.4, 0.6, 0.8, 1.0 V) on (**a**) uncoated Ti-6Al-4V; (**b**) commercially pure Ta and (**c**) the Ta_2_N coating in Ringer’s physiological solution.

Figure 13 shows the reciprocal of the square of the *C_sc_* as a function of the applied potential for the passive films formed on the three tested specimens after being polarized at different film formation potentials (*E_f_*) for 60 min in Ringer’s physiological solution at 37 °C. It can be seen that the Mott-Schottky plots can be decomposed into two linear regions over the entire potential range, and the slopes of two linear regions in each plot are always positive, which is characteristics of n-type semiconductors behavior. Such two linear regions located at low and high potentials have also been reported for other metal oxides in different media [46,47,48]. This phenomenon was attributed to a transition of passive film character from semiconducting behavior to dielectric behavior, as discussed in our recent work [49]. Furthermore, it can be seen from Figure 13 that the magnitude of *C_sc_*^−2^ and the slopes of *C_sc_*^−2^ vs. *E* increase with increasing the *E_f_*, denoting that the donor densities (*N_d_*) decrease with an increase in *E_f_*. These accords with theoretical prediction based on the point defect model (PDM) [50]. Schmidt et al. [51] suggested that the higher *E_f_* was favorable for the formation of a highly ordered and denser passive film, accompanied by a reduction in carrier density. As shown in Figure 13b, it is important to note that at the *E_f_* values of 0.6, 0.8 and 1.0 V, the negative slopes of the straight lines are observed for commercially pure Ta at potentials above 0.3 V, which is attributed to the formation of an inversion layer derived from an increase in hole concentration in the valence band [52].

According to Equation (7), the calculated *N_d_* and *E_fb_* values for the passive films formed on the three tested samples at different potentials in Ringer’s physiological solution at 37 °C are summarized in Table 3. As shown in Table 3, with increasing the *E_f_*, the *N_d_* decreases and the *E_fb_* moves slightly to more positive potential for all of the tested samples. At a given *E_f_*, values of *N_d_* obtained from the passive film on the Ta_2_N coating are in the range of 10^18^–10^19^ cm^−3^, which are substantially lower than those for the passive films formed on both uncoated Ti-6Al-4V and commercially pure Ta. It is known that the larger *N_d_* represents the higher conductivity of the passive film, which will result in an increase in the passive current density and reduce the protective effect of the passive film. From the electronic standpoint, lower *N_d_* in the passive film on the Ta_2_N coating restrains the transfer of the electron and impedes the electrochemical reaction in the passive film. This results account for the different passive current density of the three tested samples shown in Figure 8 [53,54]. Moreover, the presence of aggressive chloride ions (Cl^−^) in Ringer’s physiological solution will exert a detrimental effect on the stability of the passive film. According to the point defect model (PDM) proposed by Macdonald [50], chloride ions can be absorbed into oxygen vacancies at the passive film/solution interface. The absorption of chloride ions into the oxygen vacancies rise the local cation vacancy concentration and then enhances the electromigration-dominated flux of cation vacancies from the barrier layer/solution interface to the metal or coating/barrier layer interface, where they are annihilated by an oxidative injection of cation from the metal or coating into the film. If the annihilation reaction cannot accommodate the enhanced flux of cation vacancies, the excess vacancies will condense to form voids, causing a collapse of the film. Hence, compared with those on the uncoated Ti-6Al-4V and commercially pure Ta, the decreased oxygen vacancies of the passive film on the Ta_2_N coating can lower the cation vacancy concentration at the metal or coating/barrier layer interface and enhance the stability of the passive film.

The thickness of the space-charge layer (*δ_sc_*) in a semiconductor is an important parameter that controls the movement of electrons. The space charge layer thickness (*δ_sc_*) for an n-type semiconductor can be calculated from the following equation [55]:

(8) $$\delta_{sc}={\left[\frac{2\varepsilon_{r}\varepsilon_{0}}{eN_{d}}\left(E-E_{fb}-\frac{kT}{e}\right)\right]}^{1/2}$$

As shown in Table 3, at a given *E_f_*, the Ta_2_N coating has the largest *δ_sc_*, while commercially pure Ta has the lowest. Because the space charge layer thickness scales with the thickness of the oxide layer, thicker passive films are less susceptible to breakdown and pitting, and provide higher protection against corrosion [56]. Thereby, it is reasonable to conclude that, under identical passivation conditions, the thickness of the passive film grown on the tested specimens increases in the order of commercially pure Ta < uncoated Ti-6Al-4V < Ta_2_N coating.

**Figure 14:**
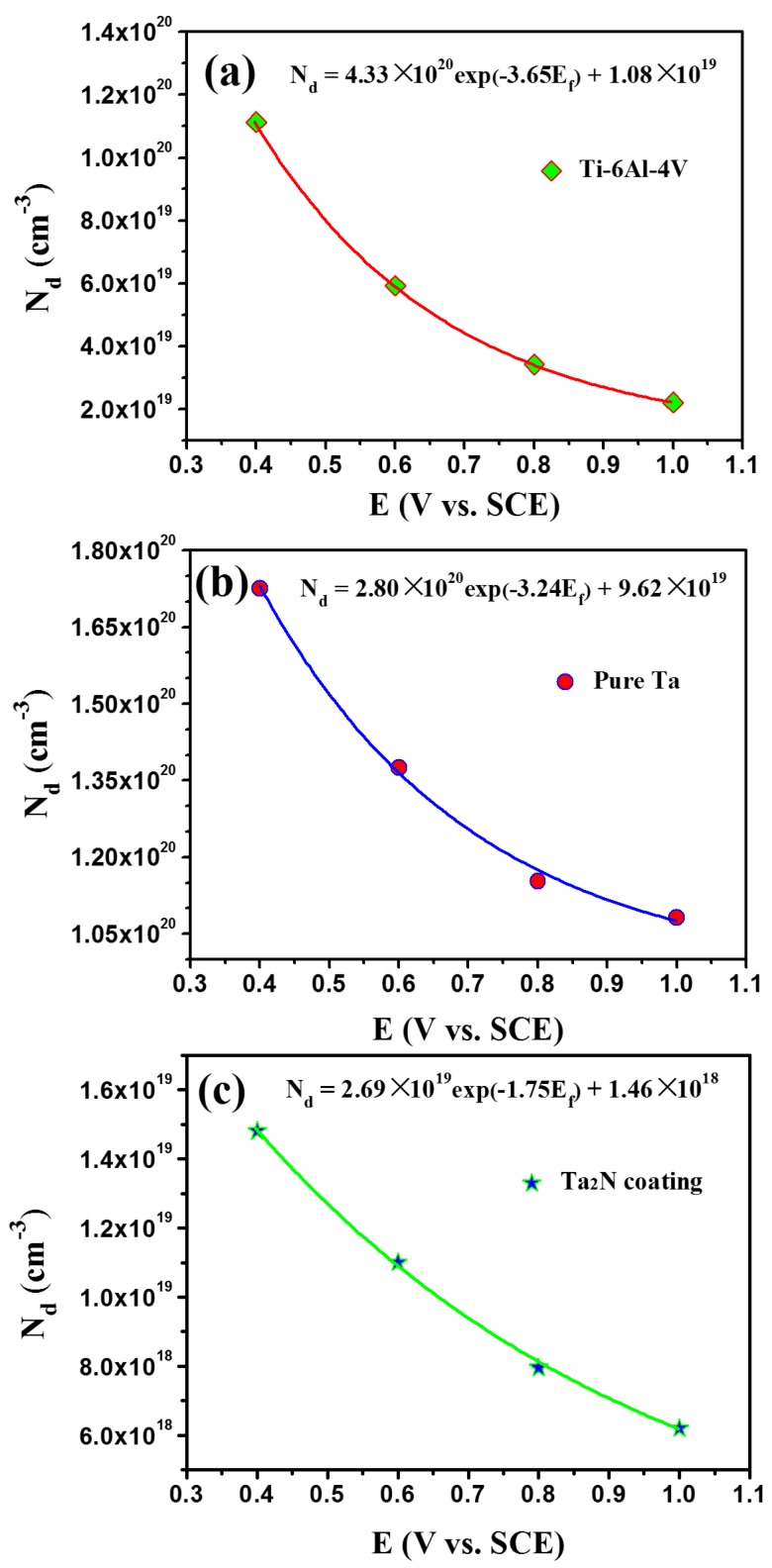
Donor density (*N_d_*) in the passive films formed on (**a**) uncoated Ti-6Al-4V; (**b**) commercially pure Ta and (**c**) the Ta_2_N coating in Ringer’s physiological solution as a function of film formation potential (*E_f_*). The solid lines are the exponential fit of N_d_ on E_f_.

The flux of the point defects is essential to the growth and breakdown of the passive film. The diffusivity (*D_o_*) qualitatively describes the transport of the point defects in the passive film from a microscopic perspective based on the PDM. It is reported that the field strength of the passive film can reach a magnitude of approximately 10^6^ V/cm and, under a high field strength condition, the diffusivity (*D_o_*) can be calculated by Equation (9) [46,57]:

(9) $$D_{o}=\frac{z\alpha i_{ss}}{e\omega_{2}\exp\left(z\alpha \bar{\varepsilon }F/RT\right)}$$

where *z* is the charge number of the mobile point defects in the passive film (*z* = 2 is used in this work), *α* is the half-jump distance of migrating point defects, assuming *α* = 0.25 nm [57], *i_ss_* is the steady state passive current density, which can be determined from the polarization curves, *e* is the charge of an electron (1.6 × 10^−^^19^ C), *ω*_2_ is a unknown constant that can be acquired by exponentially fitting the *N_d_*-*E_f_* plot (shown in Figure 14), $\bar{\varepsilon }$ is the mean field strength of the passive film (approximately 3.0 × 10^6^ V/cm [58]), *F* is the Faraday constant (96.485 C/mol), *R* is the gas constant (8.314 J/K·mol), and *T* is the temperature in Kelvin (310 K).

In order to obtain the value of *ω*_2_, the dependence of *N_d_* on *E_f_* was fitted using a first-order exponential decay function [46,50,57]:

(10) $$N_{d}=\omega_{1}exp\left(-bE_{f}\right)+\omega_{2}$$

The *N_d_* of the passive films formed on the tested specimens under different *E_f_* and the corresponding exponential fitted results are shown in Figure 14. The values of *ω*_2_ for the passive films formed on uncoated Ti-6Al-4V, commercially pure Ta and the Ta_2_N coating are 1.08 × 10^19^ cm^−^^3^, 9.62 × 10^19^ cm^−3^ and 1.50 × 10^18^ cm^−3^, respectively. Then, substituting the variables *i_ss_*, *ω*_2_, z, e, $\bar{\varepsilon }$, F, R, T, and *α* into Equation (9) to calculate the diffusivity of the point defects in the passive films, which are listed in Table 3. The calculated *D_o_* value for the Ta_2_N coating (1.94 × 10^−16^ cm^2^/s) is comparable to that for commercially pure Ta (1.13 × 10^−16^ cm^2^/s), both of which are one order of magnitude less than that for uncoated Ti-6Al-4V (2.73 × 10^−15^ cm^2^/s). As a result, it can be concluded that the lower *N_d_*, combined with the lower *D_o_* for the Ta_2_N coating, impedes the electrochemical reaction from occurring on its passive film and improves the stability of the passive film. This enhances the protective ability of the Ta_2_N coating. Moreover, it can be noted here that during the anodic polarization of the Ta_2_N coating, the formation of ammonium ions consumes protons that raises the local pH [59], reducing the acidifying effect of metal dissolution. The NH_4_^+^ ions may possibly undergo further reaction to the formation of stable NO_x_^−^ species, such as NO_2_^−^ or NO_3_^−^, which can further improve resistance to pitting through repelling the adsorption of chloride ions on the film/electrolyte interface. By the way, compared with uncoated Ti-6Al-4V, commercially pure Ta has a higher donor density and thinner passive film, yet it shows a higher corrosion resistance in Ringer’s physiological solution, mainly originating from its lower *D_o_*.

## 4. Conclusions

In this study, a Ta_2_N nanoceramic coating was fabricated onto Ti-6Al-4V substrate in an Ar and N_2_ gas mixture using a double glow discharge plasma process. The as-deposited coating consists of fine nanocrystals with an average grain size of 12.8 nm. The coating enhances the surface hardness of Ti-6Al-4V and exhibits good contact damage tolerance and sufficient adhesion strength to meet requirements for long-term use in the human body under load-bearing conditions. The corrosion resistance of the Ta_2_N coating was investigated by various electrochemical analytical techniques in Ringer’s physiological solution at 37 °C, and was compared to commercially pure Ta and uncoated Ti-6Al-4V. Potentiodynamic polarization showed that the Ta_2_N coating could be passivated spontaneously in Ringer’s physiological solution and showed a more positive *E_corr_* and lower i_corr_ than both commercially pure Ta and uncoated Ti-6Al-4V. EIS results indicated that the resistance value (R_p_) of the Ta_2_N coating was three times greater than that of commercially pure Ta, and was one order of magnitude larger than that of uncoated Ti-6Al-4V. The Mott-Schottky analysis indicated that the passive film formed on the Ta_2_N coating are in the range of 10^18^–10^19^ cm^−3^, which are substantially lower than those for the passive films formed on both commercially pure Ta and uncoated Ti-6Al-4V. In light of its high electrochemical stability combined with good mechanical properties, the Ta_2_N coating has potential for the surface protection of metallic orthopedic devices.

## Figures and Tables

**Table 1:** Electrochemical parameters extracted from potentiodynamic curves of investigated specimens in Ringer’s physiological solution at 37 °C.

Samples	Ti-6Al-4V	Pure Ta	Ta_2_N Coating
*E_corr_* (V vs. SCE)	−0.24	−0.21	−0.12
*β*_a_ (mV/decade)	158.08	249.25	305.16
-*β*_c_ (mV/decade)	116.48	118.04	120.63
*i*_corr_ (A·cm^−2^)	4.20 × 10^−7^	2.99 × 10^−7^	6.76 × 10^−9^
*i*_pass_ **^1^** (A·cm^−2^)	9.86 × 10^−6^	3.63 × 10^−6^	4.55 × 10^−8^
*R_p_* (Ω·cm^2^)	6.93 × 10^4^	1.16 × 10^5^	5.55 × 10^6^
*p* (%)	–	–	0.22

**^1^** The passive current densities were derived at 0.6 V vs. SCE.

**Table 2:** Electrochemical parameters derived from impedance fitting for investigated specimens at their respective open circuit potentials in Ringer’s physiological solution at 37 °C.

Samples	Ti-6Al-4V	Pure Ta	Ta_2_N Coating
*R_s_* (Ω·cm^2^)	26.25 ± 0.17	25.21 ± 0.20	34.74 ± 0.35
*Q_p_* (Ω^−1^·cm^−2^·s^n^)	(1.15 ± 0.01) × 10^−^^5^	(1.26 ± 0.01) × 10^−^^5^	(5.72 ± 0.05) × 10^−^^6^
n	0.911 ± 0.001	0.919 ± 0.001	0.941 ± 0.002
*R_p_* (Ω·cm^2^)	(5.75 ± 0.15) × 10^5^	(1.21 ± 0.04) × 10^6^	(4.70 ± 0.31) × 10^6^
*C_p_* (μF·cm^−2^)	8.43	6.19	3.35
*τ* (s)	4.85	7.49	15.75
*χ*^2^	9.35 × 10^−4^	5.61 × 10^−4^	6.33 × 10^−4^

**Table 3:** Summary of parameters derived from capacitance measurements and PDM analysis for the passive films formed on the investigated specimens in Ringer’s physiological solution.

Samples		Ti-6Al-4V	Pure Ta	Ta_2_N Coating
0.4 V	*N_d_* (×10^19^ cm^−3^)	11.13	17.26	1.48
	*E_fb_* (V)	−1.15	−1.66	−1.50
	*δ_sc_* (nm)	9.61	5.74	18.85
0.6 V	*N_d_* (×10^19^ cm^−3^)	5.92	13.75	1.10
	*E_fb_* (V)	−0.90	−1.57	−1.49
	*δ*_sc_ (nm)	12.96	6.60	22.93
0.8 V	*N_d_* (×10^19^ cm^−3^)	3.42	11.53	0.80
	*E_fb_* (V)	−0.80	−1.53	−1.47
	*δ*_sc_ (nm)	17.61	7.47	28.02
1.0 V	*N_d_* (×10^19^ cm^−3^)	2.20	10.81	0.62
	*E_fb_* (V)	−0.78	−1.52	−1.48
	*δ*_sc_ (nm)	23.16	8.02	33.27
*ω*_2_ (×10^19^ cm^−3^)		1.08	9.62	0.15
*i_ss_* (×10^−6^A·cm^−2^)		9.50	3.49	0.10
*D_o_* (×10^−16^ cm^2^/s)		27.33	1.13	1.94

## References

[1] Kurtz S., Ong K., Lau E., Mowat F., Halpem M. (2007). Projections of primary and revision hip and knee arthroplasty in the United States from 2005 to 2030. J. Bone Jt. Surg. Am..

[2] Geetha M., Singh A.K., Asokamani R., Gogia A.K. (2009). Ti based biomaterials, the ultimate choice for orthopaedic implants—A review. Prog. Mater. Sci..

[3] Hanawa T. (2004). Metal ion release from metal implants. Mater. Sci. Eng. C.

[4] Sun Z.L., Wataha J.C., Hanks C.T. (1997). Effects of metal ions on osteoblast-like cell metabolism and differentiation. Biomed. J. Mater. Res..

[5] Rao S., Ushida T., Tateishi T., Okazaki Y., Asao S. (1996). Effect of Ti, Al and V ions on the relative growth rate of fibroblasts (L929) and osteoblasts (MC3T3-E1) cells. Bio-Med. Mater. Eng..

[6] Niinomi M. (2003). Recent research and development in titanium alloys for biomedical applications and healthcare goods. Sci. Technol. Adv. Mater..

[7] Hallab N.J., Jacobs J.J. (2003). Orthopedic implant fretting corrosion. Corros. Rev..

[8] Liu X., Chu P.K., Ding C. (2004). Surface modification of titanium, titanium alloys, and related materials for biomedical applications. Mater. Sci. Eng. R.

[9] Ching H.A., Choudhury D., Nine M.J., Osman N.A.A. (2014). Effects of surface coating on reducing friction and wear of orthopaedic implants. Sci. Technol. Adv. Mater..

[10] Liu L.L., Xu J., Munroe P., Xu J.K., Xie Z.-H. (2014). Electrochemical behavior of (Ti_1__–x_Nb_x_)_5_Si_3_ nanocrystalline films in simulated physiological media. Acta Biomater..

[11] Xu J., Hu W., Xu S., Munroe P., Xie Z.-H. (2016). Electrochemical properties of a novel *β*-Ta_2_O_5_ nanoceramic coating exposed to simulated body solutions. ACS Biomater. Sci. Eng..

[12] Zhang Q.Y., Mei X.X., Yang D.Z., Chen F.X., Ma T.C., Wang Y.M., Teng F.N. (1997). Preparation, structure and properties of TaN and TaC films obtained by ion beam assisted deposition. Nucl. Instrum. Methods Phys. Res. B.

[13] Tsai M.H., Sun S.C., Lee C.P., Chiu H.T., Tsai C.E., Chuang S.H., Wu S.C. (1995). Metal-organic chemical vapor deposition of tantalum nitride barrier layers for ULSI applications. Thin Solid Films.

[14] Lovejoy M.L., Patrizi G.A., Roger D.J., Barbour J.C. (1996). Thin-film tantalum-nitride resistor technology for phosphide-based optoelectronics. Thin Solid Films.

[15] Leng Y.X., Sun H., Yang P., Chen J.Y., Wang J., Wan G.J., Huang N., Tian X.B., Wang L.P., Chu P.K. (2001). Biomedical properties of tantalum nitride films synthesized by reactive magnetron sputtering. Thin Solid Films.

[16] Oliver W.C., Pharr G.M. (1992). An improved technique for determining hardness and elastic modulus using load and displacement sensing indentation experiments. J. Mater. Res..

[17] Jiang H.G., Rühle M., Lavernia E.J. (1999). On the applicability of the X-ray diffraction line profile analysis in extracting grain size and microstrain in nanocrystalline materials. J. Mater. Res..

[18] Moody N.R., Medlin D., Boehme D., Norwood D.P. (1998). Film thickness effects on the fracture of tantalum nitride on aluminum nitride thin film systems. Eng. Fract. Mech..

[19] Nie X., Leyland A., Matthews A. (2000). Deposition of layered bioceramic hydroxyapatite/TiO_2_ coatings on titanium alloys using a hybrid technique of micro-arc oxidation and electrophoresis. Surf. Coat. Technol..

[20] Hogmark S., Jacobson S., Larsson M. (2000). Design and evaluation of tribological coatings. Wear.

[21] Munoz A.I., Mischler S. (2007). Interactive effects of albumin and phosphate ions on the corrosion of CoCrMo implant alloy. J. Electrochem. Soc..

[22] Chidambaram D., Clayton C.R., Dorfman M.R. (2004). Evaluation of the electrochemical behavior of HVOF-sprayed alloy coating. Surf. Coat. Technol..

[23] Starosvetsky D., Gotman I. (2001). Corrosion behavior of titanium nitride coated Ni-Ti shape memory surgical alloy. Biomaterials.

[24] Creus J., Mazille H., Idrissi H. (2000). Porosity evaluation of protective coatings onto steel, through electrochemical techniques. Surf. Coat. Technol..

[25] Stern M., Geary A.L. (1957). Electrochemical polarization I. A theoretical analysis of the shapes of polarization curves. J. Electrochem. Soc..

[26] Pacha-Olivenza M.A., Gallardo-Moreno A.M., Vadillo-Rodríguez V., González-Martín M.L., Péres-Giraldo C., Galván J.C. (2013). Electrochemical analysis of the UV treated bactericidal Ti6Al4V surfaces. Mater. Sci. Eng. C.

[27] Córdoba-Torres P., Mesquita T.J., Devos O., Tribollet B., Roche V., Nogueira R.P. (2012). On the intrinsic coupling between constant-phase element parameters *α* and Q in electrochemical impedance spectroscopy. Electrochim. Acta.

[28] Potucek R.K., Rateick R.G., Birss V.I. (2006). Impedance characterization of anodic barrier Al oxide film beneath porous oxide layer. J. Electrochem. Soc..

[29] Brug G.J., van den Eeden A.L.G., Sluyters-Rehbach M., Sluyters J.H. (1984). The analysis of electrode impedances complicated by the presence of a constant phase element. J. Electroanal. Chem..

[30] Jiang P., Lin L., Zhang F., Dong X., Ren L., Lin C. (2013). Electrochemical construction of micro-nano spongelike structure on titanium substrate for enhancing corrosion resistance and bioactivity. Electrochim. Acta.

[31] Gray J.J., Orme C.A. (2007). Electrochemical impedance spectroscopy study of the passive films of alloy 22 in low pH nitrate and chloride environments. Electrochim. Acta.

[32] Labjar N., Lebrini M., Bentiss F., Chihib N.-E., Hajjaji S.E., Jama C. (2010). Corrosion inhibition of carbon steel and antibacterial properties of aminotris-(methylenephosphonic) acid. Mater. Chem. Phys..

[33] Macdonald D.D., Urquidi-Macdonald M. (1990). Theory of steady-state passive films. J. Electrochem. Soc..

[34] Lakatos-Varsányi M., Falkenberg F., Olefjord I. (1998). The influence of phosphate on repassivation of 304 stainless steel in neutral chloride solution. Electrochim. Acta.

[35] Chang C.-C., Jeng J.S., Chen J.S. (2002). Microstructural and electrical characteristics of reactively sputtered Ta-N thin films. Thin Solid Films.

[36] Lamour P., Fioux P., Ponche A., Nardin M., Vallat M.-F., Dugay P., Brun J.-P., Moreaud N., Pinvidic J.-M. (2008). Direct measurement of the nitrogen content by XPS in self-passivated TaN_x_ thin films. Surf. Interface Anal..

[37] Atanassova E., Spassov D. (1998). X-ray photoelectron spectroscopy of thermal thin Ta_2_O_5_ films on Si. Appl. Surf. Sci..

[38] Olefjord I., Wegrelius L. (1996). The fluence of nitrogen on the passivation of stainless steels. Corros. Sci..

[39] Chun W.-J., Ishikawa A., Fujisawa H., Takata T., Kondo J.N., Hara M., Kawai M., Matsumoto Y., Domen K. (2003). Conduction and valence band positions of Ta_2_O_5_, TaON, and Ta_3_N_5_ by UPS and electrochemical methods. J. Phys. Chem. B.

[40] Escrivà-Cerdán C., Blasco-Tamarit E., García-García D.M., García-Antón J., Guenbour A. (2012). Effect of potential formation on the electrochemical behaviour of a highly alloyed austenitic stainless steel in contaminated phosphoric acid at different temperatures. Electrochim. Acta.

[41] Jovic V.D., Barsoum M.W. (2004). Corrosion behavior and passive film characteristics formed on Ti, Ti_3_SiC_2_, and Ti_4_AlN_3_ in H_2_SO_4_ and HCl. J. Electrochem. Soc..

[42] Morrison S.R. (1980). Electrochemistry at Semiconductor and Oxidized Metal Electrodes.

[43] Kerrec O., Devilliers D., Groult H., Chemla M. (1995). Dielectric properties of anodic films on tantalum. Electrochim. Acta.

[44] Milošev I., Metikoš-Huković M., Strehblow H.-H. (2000). Passive film on orthopaedic TiAlV alloy formed in physiological solution investigated by X-ray photoelectron spectroscopy. Biomaterials.

[45] Martini E.M.A., Muller I.L. (2000). Characterization of the film formed on iron in borate solution by electrochemical impedance spectroscopy. Corros. Sci..

[46] Li D.G., Wang J.D., Chen D.R. (2012). Influence of potentiostatic aging, temperature and pH on the diffusivity of a point defect in the passive film on Nb in an HCl solution. Electrochim. Acta.

[47] Jović V.D., Jović B.M. (2008). Properties of ZrO_2_ passive film formed onto Zr electrode in 1 m NaOH at low voltage. J. Electrochem. Soc..

[48] Li D.G. (2015). Effect of ultrasonic cavitation on the diffusivity of a point defect in the passive film on formed Nb in 0.5 M HCl solution. Ultrason. Sonochem..

[49] Liu L.L., Xu J., Lu X.L., Munroe P., Xie Z.-H. (2016). Electrochemical corrosion behavior of nanocrystalline *β*-Ta coating for biomedical applications. ACS Biomater. Sci. Eng..

[50] Macdonald D.D. (1999). Passivity–the key to our metals-based civilization. Pure Appl. Chem..

[51] Schmidt A.M., Azambuja D.S., Martini E.M.A. (2006). Semiconductive properties of titanium anodic oxide films in McIlvaine buffer solution. Corros. Sci..

[52] Harrington S.P., Devine T.M. (2009). Impedance study of alloy 22 in hydrochloric acid using a semiconductor model. ECS Trans..

[53] Katkar V.A., Gunasekaran G., Rao A.G., Koli P.M. (2011). Effect of the reinforced boron carbide particulate content of AA6061 alloy on formation of the passive film in seawater. Corros. Sci..

[54] Kang J., Yang Y., Jiang X., Shao H. (2008). Semiconducting properties of passive films formed on electroplated Ni and Ni-Co alloys. Corros. Sci..

[55] Metikoš-Huković M., Grubač Z. (1998). Characterization of electronic and dielectric properties of anodic oxide films on bismuth by electrochemical impedance spectroscopy. J. Phys. Chem. B.

[56] Martin F.J., Cheek G.T., O’Grady W.E., Natishan P.M. (2005). Impedance studies of the passive film on aluminium. Corros. Sci..

[57] Kong D.-S., Lu W.-H., Feng Y.-Y., Yu Z.-Y., Wu J.-X., Fan W.-J., Liu H.-Y. (2009). Studying on the point-defect-conductive property of the semiconducting anodic oxide films on titanium. J. Electrochem. Soc..

[58] Macdonald D.D. (2011). The history of the Point Defect Model for the passive state: A brief review of film growth aspects. Electrochim. Acta.

[59] Jargelius-Pettersson R.F.A. (1999). Electrochemical investigation of the influence of nitrogen alloying on pitting corrosion of austenitic stainless steels. Corros. Sci..

